# Isolation and characterization of TayeBlu, a novel bacteriophage of *Azotobacter vinelandii*

**DOI:** 10.1128/spectrum.01286-25

**Published:** 2025-12-09

**Authors:** Taiwo Mercy Akanbi, Mariana Labat, Tyler Sun, Derek A. Smith, Sarah C. Bagby

**Affiliations:** 1 Department of Biology, Case Western Reserve University 228363, Cleveland, Ohio, USA; Centre de Biologie Integrative, Toulouse, France

**Keywords:** bacteriophages, soil microbiology, diazotrophs

## Abstract

Soil microbial communities drive global biogeochemical cycles and alter crop yields through nitrogen fixation. As agents of genetic mobility, mortality, and nutrient release, viruses have been shown to influence microbial community structure and activity in numerous marine and aquatic systems. However, their impacts on terrestrial ecosystems are less well understood, in part because few model phage-host systems have been established for soils. To fill this gap, we sought to develop a new model system for viral infection of nitrogen-fixing bacteria derived from agricultural soil. Here, we report the isolation, characterization, and sequencing of the novel bacteriophage TayeBlu, which infects the globally distributed aerobic soil bacterium *Azotobacter vinelandii*, a facultative diazotroph. TayeBlu was isolated from the rhizosphere of tomato plants in a farm greenhouse. We find that the availability of nitrogen to host cells strongly influences TayeBlu infection physiology at the level of adsorption kinetics, time to lysis, and burst size. Taxonomic and comparative genome analyses reveal that TayeBlu belongs to an understudied family in class *Caudoviricetes* in which a small core of structural and assembly genes has persisted through adaptive diversification on different bacterial hosts.

**IMPORTANCE:**

Understanding the forces regulating soil microbial activity is critical for building accurate ecosystem models that can inform land-management strategies for mitigating climate risks and stabilizing global food supply. For agricultural sustainability, it is particularly important to understand the dynamics of nitrogen-fixing soil bacteria like *Azotobacter vinelandii*, a well-studied and globally distributed species whose activity promotes plant growth and soil fertility. To support detailed investigations of the impact of viruses on diazotroph ecosystem outputs, we isolated and investigated a novel soil virus that infects *A. vinelandii*. The resulting new phage-host system expands the limited toolkit of model experimental systems for soil viral studies. By enabling investigations of viral impacts on terrestrial nitrogen-fixing bacteria, this work sets the stage for future studies illuminating a critical but poorly understood aspect of soil ecology. Moreover, TayeBlu belongs to a novel viral family, and this study provides the first description of this family’s conserved features.

## INTRODUCTION

Soil microbial communities shape global biogeochemical cycles and local ecosystem function through metabolic processes and ecological interactions that determine the bioavailability of carbon (C) and nitrogen (N) (1, 2), affecting plant productivity and soil function. Within these communities, N-fixing bacteria (diazotrophs) play a crucial role by converting atmospheric N_2_ into biologically available forms. Annually, biological N fixation contributes ∼200 million metric tons of bioavailable N to Earth’s ecosystems (3, 4), including nearly half the total N present in crop fields (5, 6). By releasing organic N as a public good, soil diazotrophs facilitate the growth of plants and other microbes (7, 8), which otherwise may scavenge N by degrading recalcitrant soil organic matter (7, 8), potentially increasing carbon dioxide emissions (9).

Infection of bacteria by their viruses (bacteriophages, or phages) alters host ecological impacts (10). Phages influence nutrient cycling and control bacterial population dynamics and mortality (11). In addition to direct lysis, phage infection impacts bacterial communities and biogeochemical processes through several distinct mechanisms: phages can (i) reprogram host metabolism by introducing auxiliary metabolic genes (AMGs) (12, 13); (ii) accelerate nutrient turnover through release of host cellular contents (14, 15); and (iii) transfer genetic material between hosts (16–18). Phage infection dynamics can be highly dependent on host physiological state, with low-nutrient conditions reducing phage infectivity and burst size (e.g., see references 19 and 20). Viral lysis accounts for substantial bacterial mortality and subsequent nutrient release in well-studied marine systems (14, 17, 21). While available evidence suggests that soil environments contain substantial phage populations (∼10^3^ to 10^9^ viral particles per gram of soil, depending on soil type and conditions [22]), our knowledge of phage-host dynamics in soil ecosystems lags considerably behind that in marine systems (23, 24).

A major challenge in soil virology is the limited number of established phage-host systems available for experimental investigation, with current models capturing only a handful of the predominant soil bacterial phyla (25, 26). This limitation is especially pronounced for N-fixing bacteria, a problematic gap given the critical importance of diazotrophy for soil function. In particular, the observation that N flow to the soil system can differ substantially between diazotrophs (e.g., between plant-associated and free-living species) (27) points to a need for diverse experimental systems to support comparative analysis of the ecosystem impacts of phage infection of different diazotrophic hosts. Recent work has identified phages infecting *Klebsiella* sp. M5al (28), a facultative diazotroph from a genus that often grows in association with a plant host. *Klebsiella* spp. fix N anaerobically, with low levels of O_2_ rapidly inhibiting nitrogenase, the enzyme responsible for N fixation (29). By contrast, the globally distributed soil bacterium *Azotobacter vinelandii* is a free-living and obligately aerobic facultative diazotroph (30, 31). Azotobacters are noteworthy for their specialized metabolic adaptations (e.g., extraordinarily high respiration rates) to protect nitrogenase from oxygen (reviewed in reference 30), offering an opportunity to investigate infection physiology under widely different host metabolic states. With a versatile metabolism, a long history as a soil model system, and modern tools for mechanistic investigation (32, 33), *A. vinelandii* is an ideal host for phage model system development. Despite numerous reports of characterized *A. vinelandii* phage isolates several decades ago (34–36), to our knowledge, none of these phages remain in cultivation, and none have been sequenced or characterized by modern methods.

To fill this gap, we sought to isolate a novel phage of *A. vinelandii* that can be developed as an experimental model system for investigation of phage impacts on free-living soil diazotrophs. Here, we report the isolation, characterization, and sequencing of the novel rhizosphere siphovirus TayeBlu infecting *A. vinelandii* strain OP, and we demonstrate that phage infection is significantly influenced by N availability in the medium. Through comprehensive genomic analysis, we identify this phage as belonging to a novel viral family within an unclassified order of the class *Caudoviricetes*, with a conserved core of structural and replication genes but low genomic similarity to other known soil phages. This novel phage shows promise as an experimental system for studies on the influence of soil phages on N-fixing bacterial communities in variable soil environments and their impact on global biogeochemical cycles (23).

## MATERIALS AND METHODS

### Host strain, growth media, and phage buffer

All experiments were performed with *Azotobacter vinelandii* strain OP (hereafter *A. vinelandii*), which was the generous gift of Xinning Zhang. *A. vinelandii* was grown in three media spanning high and low C and N availabilities. Peptone-yeast-calcium (PYCa) medium is a rich and N-replete medium consisting of 15 g L^−1^ peptone, 1 g L^−1^ yeast extract, 1 g L^−1^ dextrose, and 4.5 mM CaCl_2_. Dean’s Burke (Dean) medium is a minimal, N-free medium consisting of 1 mM phosphate buffer, 20 g L^−1^ sucrose, 0.8 mM MgSO_4_, 0.6 mM CaCl_2_, 1 µM Na_2_MoO_4_, and 18 µM FeSO_4_. As an intermediate condition, we supplemented Dean with ammonium chloride (1 mM NH_4_Cl final concentration) as an N source (AC_Dean). Phage buffer consisted of 68 mM NaCl, 1 mM CaCl_2_, 10 mM MgSO_4_, and 10 mM Tris, pH 7.5.

### Isolation and purification of phage TayeBlu

Phage TayeBlu (named from “Taye,” a Nigerian-Yoruba name for “first child to taste the world,” reflecting its status as the first sequenced and genomically characterized *A. vinelandii* phage isolate, and “Blu,” a local reference reflecting the isolate’s Cleveland origin) was isolated from rhizosphere soil collected at the base of a tomato plant in a greenhouse at the Case Western Reserve University Farm, Hunting Valley, OH (41°29′36″N, 81°25′24″W), on 30 August 2023. Using a hand shovel sterilized with 70% ethanol, 50–55 g of dry, dark soil was collected from within 2 cm of the plant base (Fig. S1), where fine roots were visible. Ambient temperature was 18°C. Samples were stored on ice and kept at 4°C until further processing.

Phage isolation used the enrichment method (37), followed by spot test analysis and agarose overlays (38). Briefly, fresh soil samples (∼25 g) were enriched with 500 μL of an overnight *A. vinelandii* culture and incubated at 30°C for 48 h with shaking at 225 rpm. The mixture was centrifuged at 4,700 × *g* for 30 min, and the supernatant was passed through a 0.22-µm pore-size polyethersulfone (PES) filter to remove bacterial cells. Filtrates were screened for phage activity using the spot test method (39) with *A. vinelandii* cultured in the three media described above. Briefly, 500 μL of overnight culture was mixed with 4.5 mL of 0.6% (wt/vol) molten agar and spread evenly on a 1.6% (wt/vol) agar plate. After solidification of the top agar matrix, 10 μL of filtrate was spotted onto the surface and allowed to dry. Plates were incubated at 30°C for 24 h and monitored for plaque formation. The presence of a clear or turbid clearing on the plated bacterial lawn was considered a putative phage plaque.

Putative phage plaques were cored and suspended in 100 μL phage buffer, serially diluted, and then plated using the agarose overlay method. Ten microliters of the target dilution (typically 10^−4^ to 10^−6^) was added to 500 μL overnight culture and 4.5 mL of 0.6% (wt/vol) molten agar and spread evenly on a 1.6% (wt/vol) agar plate. Plates were incubated at 30°C overnight. For three consecutive rounds of purification, a single, isolated plaque was re-suspended in 100 μL of phage buffer, diluted, and plated to obtain a clonal phage population. Purified clonal phage was then amplified, filtered (0.22-μm pore-size PES filters), titered, and stored (4°C for immediate use, −80°C in 6.54% dimethyl sulfoxide or 20% glycerol for freezer stock) (39). Only stocks stored at 4°C were used for further characterization in this study.

### Phage-host adsorption kinetics

To determine the adsorption rate constants (k) of phage TayeBlu and *A. vinelandii* strain OP in PYCa, Dean, and AC_Dean media, we performed adsorption assays following previously described protocols (40, 41). Briefly, in each medium, triplicate cultures of *A. vinelandii* were grown to mid-exponential phase (∼10^8^ colony-forming units [CFU] mL^−1^) as determined by optical density (OD) measurements and OD-to-CFU calibration curves established previously for each medium. An aliquot of 2 × 10^8^ cells was removed from each culture and transferred to a five-mL glass culture tube, and the volume was adjusted to 2 mL with fresh medium to achieve a culture density of 1 × 10^8^ CFU mL^−1^. High-titer TayeBlu lysate (4.3–7.3 × 10^10^ PFU mL^−1^) was added to a multiplicity of infection (MOI) of 0.1.

Immediately following infection ($t_{0}$) and at pre-determined intervals, samples were collected for both total and free phage quantification. For free phage measurements, samples were immediately filtered through 0.22-µm pore-size PES filters to remove bacterial cells and any cell-associated phages. For total phage measurements, parallel samples were left unfiltered. All samples were serially diluted in phage buffer and enumerated at two sequential dilutions using the agarose overlay method. AC_Dean was used as the base medium for plaque assays from AC_Dean adsorption experiments; PYCa was used for plaque assays from experiments in both PYCa and Dean. This use of PYCa ensured reliable plaque formation, as our preliminary experiments demonstrated that PYCa provides consistently good conditions for TayeBlu plaque formation on this host. Plates were incubated at 30°C, and plaques were enumerated after 24, 48, and 72 h to ensure complete development of all viable plaques.

Statistical analyses of the decrease in free phage concentration over time were performed using R (v.4.4.3) (42) with the tidyverse (43), Hmisc (44), errors (45), ggtext (46), BSDA (47), and patchwork (48) packages. Data were filtered to exclude dilutions with zero counts and those with plaques too numerous to count (TNTC). For each measurement, the phage concentration (plaque-forming units [PFU] per milliliter) was calculated and assigned an uncertainty scaled to the square root of the number of plaques observed (truncated at a minimum of 1.2), reflecting Poisson error in counting statistics. Weighted means and standard deviations (SDs) were calculated for each time point, with weights assigned as the inverse of the relative error for each measurement. In cases where the weighted coefficient of variation was greater than 0.8 and at least four measurements (of the six attempted, three biological replicates × two dilutions) produced quantitative (non-zero, non-TNTC) results, we checked for influential outliers using jackknife resampling as follows. For each such measurement, we calculated the absolute values of (i) the difference between the full weighted mean and the jackknife weighted mean, relative to the jackknife weighted mean, to judge the measurement’s influence on sample mean; and (ii) the difference between the full weighted SD and the jackknife weighted SD, relative to the full weighted SD, to judge the measurement’s contribution to the sample error. We set thresholds for both quantities at 0.5 and considered measurements outliers only if they exceeded both thresholds. For adsorption experiments, no measurements were excluded as outliers.

Following reference 40, the fraction of free phage remaining ($P_{t}/P_{0}$) was plotted against time. Adsorption rate constants (k, in units of mL min^−1^) were determined by fitting the natural log-transformed data to a first-order kinetic model, $k=-\ln(P_{t}/P_{0})/Nt$, where $P_{t}$ is the concentration of free phage at time t, $P_{0}$ is the initial free phage concentration, and N is the bacterial concentration. All error propagation used the errors package. Differences in fitted k values were tested with Welch’s modified two-sample *t*-test (BSDA tsum.test).

### One-step growth curves

To determine the latent period and average burst size of phage TayeBlu and *A. vinelandii* strain OP in PYCa, AC_Dean, and Dean media, we performed one-step growth experiments following previously described protocols (40, 49, 50), with an MOI of ∼0.1 (PYCa, 0.106; AC_Dean, 0.090; Dean, 0.091) and an 11-min adsorption period. In each one-step assay, triplicate cultures were grown in the test medium to mid-logarithmic phase (1.1–1.7 × 10^8^ CFU mL^−1^) as determined by optical density measurements and OD-to-CFU calibration curves established previously for each medium. Aliquots of 1 × 10^8^ cells were removed from each culture and transferred to 1.5-mL microcentrifuge tubes, then adjusted to concentrations of ∼1 × 10^8^ CFU mL^−1^ using fresh medium. High-titer TayeBlu lysate was added to achieve the target MOI with a working volume of 1 mL for each medium during the adsorption period.

After the adsorption period, 500 μL of each replicate culture was transferred to fresh 250-mL flasks containing 49.5 mL of fresh medium pre-incubated at 30°C, diluting the phage-host mixture a hundredfold to prevent new infections. Flasks were incubated at 30°C with shaking at 225 rpm. At pre-determined intervals, samples were collected for total phage (10 μL) and free phage (400 μL filtered through 0.22-µm pore-size PES filters). Phage abundance in free and total phage samples was enumerated using plaque assays on PYCa (PYCa and Dean experiments) or AC_Dean (AC_Dean experiment). Plates were incubated at 30°C for 24 h in PYCa medium or 48 h in AC_Dean medium to allow for slower host growth in this medium. Plaque counts were stable after the incubation period. Plates were permitted to develop at room temperature for up to 24 h after incubation for additional re-examination to facilitate accurate counting of the very small plaques obtained in AC_Dean. Analysis of plaque counts, assignment of uncertainties, error propagation, and outlier identification were conducted as described for phage-host adsorption kinetics. Three measurements of total phage counts (one each from PYCa at 90 min, AC_Dean at 28 min, and AC_Dean at 90 min) were excluded as outliers. Burst size was calculated as the ratio of free phage concentration at the plateau after a single infective cycle to the initial number of infected cells, where the number of infected cells was determined by subtracting the initial free phage concentration from the initial total phage concentration. (Plaque counts in total phage measurements are the sum of plaques due to free phage and plaques due to infected cells; the latter are expected to yield one plaque per infected cell.) Differences in burst sizes were tested with Welch’s modified two-sample *t*-test (BSDA tsum.test). Latent period was determined as the time between phage adsorption and the onset of the release of phage progeny (40).

### Morphological characterization with transmission electron microscopy

Virion morphology was examined by transmission electron microscopy (TEM) following a protocol modified from reference 51. Purified high-titer phage lysate (1 mL at 1 × 10^10^ PFU mL^−1^) was centrifuged at 20,630 × *g* for 50 min. The supernatant was carefully removed and replaced with 500 μL of 0.1 M ammonium acetate. The sample was centrifuged again at 20,630 × *g* for 50 min. For visualization, 5 μL of the concentrated phage suspension was applied to a carbon/Formvar-coated copper grid (Electron Microscopy Sciences, FCF300CU50, purchased from Fisher Scientific, catalog no. 50-260-36) and allowed to adsorb for 1 min. Without removing excess liquid, 5 μL of 2% uranyl acetate (wt/vol) was added for negative staining and left for an additional minute. Excess liquid was then wicked away using filter paper, and the grid was air-dried. Grids were examined using a TECNAI SPIRIT T12 TEM operating at 100 kV. Virion head diameter and tail length measurements were made by analyzing transmission electron micrographs in ImageJ (52), using the line feature to draw a line across the heads and along the tails of individual virions. The lengths of these lines were recorded, and the average measurements were calculated.

### DNA extraction and whole-genome nanopore sequencing

High-molecular-weight DNA was extracted following a modified protocol based on reference 53 and the DNeasy PowerSoil Pro (QIAGEN, catalog no. 47014) DNA kit protocol. Briefly, phage lysate (6 mL) was treated with DNase I-RNase A (added at a ratio of 0.5 µL of 10 mg mL^-1^ stock per mL lysate, for a total of 3 μL nuclease mixture) and incubated at 37°C for 30 min to eliminate contaminating nucleic acids. Phage particles were concentrated by adding 0.5 mL of 30% PEG-8000 per mL of treated lysate, followed by overnight incubation at 4°C and centrifugation at 10,000 × *g* for 10 min. The resulting pellet was re-suspended in 300 μL of 5 mM MgSO_4_ and transferred to PowerBead Pro tubes (QIAGEN, catalog no. 47014) for capsid disruption using an MP Biomedicals FastPrep 24 bead beater (6 m s^−1^ for 30 s, repeated once). DNA purification continued according to the manufacturer’s protocol using the DNeasy PowerSoil Pro Kit. DNA was barcoded with a single barcode using the SQK-NBD114.24 Native Barcoding Kit 24 (v.14), then pooled with other separately barcoded samples for adaptor ligation (SQK-NBD114.24 Native Barcoding Kit 24 v.14) and multiplex sequencing on a MinION Mk1C long-read sequencer (model MIN-101C) with an R10.4.1 flowcell (FLO-MIN114).

### Phage genome assembly and annotation

Phage genomes were assembled using Oxford Nanopore Technology sequencing data. Raw reads were basecalled using Dorado (v.0.7.2) (54) with the super-accuracy model (dna_r10.4.1_e8.2_400bps_sup@v5.0.0) with 400 bps parameters (55). Demultiplexing was performed with the --both-ends argument, followed by adapter trimming using the same software (54). BAM files were converted to FASTQ format using Samtools (v.1.21) (56). Quality assessment of the FASTQ files was conducted using FastQC (v.0.11.9) (57), and the results were aggregated with MultiQC (v.1.9) (58). Quality trimming was implemented with Prowler (59) using the F1 option (Q-score threshold of 20) to retain the longest high-quality fragments. Assembly was performed using Metaflye (v.2.9) (60) with the --meta option, which is optimized for viral genomes, and the resulting graph was visualized with Bandage (61). Because polishing tools like Medaka may reduce assembly quality for modern Dorado super-accuracy (5 Hz SUP) basecalled data sets (62), we did not perform polishing. Genome coverage depth was assessed using coverage_histogram in CoverM (v.0.7.0) (63). The completeness and potential contamination of the assembled viral genome was assessed using CheckV with the end_to_end option (64).

Because assembly into circular contigs can be an artifact of the terminal repeats or circular permutation used in packaging many phage genomes, we used Terminus.SE to detect the genomic positions of terminus sequences from sequencing coverage patterns (65). To predict the packaging mechanism, we performed phylogenetic analysis of the large terminase protein, using ClustalW2 (66) for multiple sequence alignment of TayeBlu with 44 reference phages of known packaging mechanisms, followed by neighbor-joining tree construction with bootstrap analysis (1,000 replicates) using the boot.phylo function in the ape package in R (67). To reflect the biological packaging organization detected, the genome sequence was linearized and re-numbered starting at the site of the predicted 5′ terminus (position 3560 in the circular contig assembly). Gene positions and locus numbering reflect this curation step.

Coding sequences (CDSs) were predicted using multiple tools including GeneMark, GeneMarkS, Glimmer (v.3.02) (68), Prodigal (v.2.6.3) (69), and MetaGeneAnnotator (v.1.0) (70). Predictions were integrated using a weighted scoring system as described in reference 28, with scores calculated based on gene length, overlap, protein identification, and programming potential. Functional annotations were assigned based on significant alignment scores from BLASTP (*e*-value <10^−5^) (71), Swiss-Prot (release 2023_01) (72), HHpred (probability >90% and *e*-value <1) (73), and phold (v.0.2.0) (74), which converts protein sequences into structural tokens for comparison against >1 M phage protein structural models (75–79). Phage genome visualization was performed using the final manually curated GenBank-formatted TayeBlu sequences and a modified version of the phold python plotting script (plot.py).

Assignments were manually curated to resolve conflicts between predictions with attention to genomic context; across tools, HHpred results were weighted most heavily and phold least, in line with current levels of benchmarking support for these tools. Additional genomic features, including tRNAs, introns, and spanins, were screened for using the structural and functional phage annotation pipelines in Apollo (80). Rho-independent transcription terminators were identified using ARNold (81), which employs both RNAmotif and Erpin algorithms. Predicted terminators were manually curated based on their genomic context and structural features. Putative AMGs were screened using DRAM-v (v.1.3.0) (82) with default parameters, retaining predictions with an M flag and an auxiliary score of ≤3.

### Phage taxonomy classification

Identification of related phages and taxonomic classification followed a multi-tiered approach to ensure accuracy (83). We recruited phage sequences similar to TayeBlu at the nucleotide level by comparing the complete TayeBlu genome to the National Center for Biotechnology Information (NCBI) non-redundant (nr) nucleotide database using BLASTN. The average nucleotide identity (ANI) of the closest relative was estimated by multiplying the genome coverage by the percent identity of the hit. Next, we used ViPTree’s web interface (84) and vConTACT3 (v.3.1.3) (85) to recruit additional relatives based on similarity at the protein level to phages represented in the Virus-Host DB and Viral RefSeq (v.230), respectively. This analysis identified 44 viruses with significant protein-level similarity (genomic similarity [*S*_G_] values ≥0.020) from across currently known viral diversity, in addition to the three close relatives originally identified at the nucleotide level. One of the 44 viruses was identified as a much shorter satellite phage and excluded from further analysis. The phylogenetic tree of TayeBlu and the remaining 46 relatives was visualized using the package ggtree (v.3.10.1) in R (v.4.3.3) (42, 86). TayeBlu and its eight closest relatives were submitted to VIRIDIC (87) for pairwise genomic distance analysis. VIRIDIC output was visualized using a modified version of the original VIRIDIC R script and the dendextend package (88) for dendrogram presentation.

We used vConTACT3’s hierarchical clustering of gene-sharing networks with this set of phage genomes to place TayeBlu among the *Caudoviricetes*. To refine this prediction, we broadened our comparison set to include all *Caudoviricetes* virus species exemplars and additional isolates in the latest International Committee on the Taxonomy of Viruses (ICTV) Virus Metadata Resource (VMR) database (https://ictv.global/vmr, accessed 29 May 2025). Using the VMR MSL 40.v1 metadata sheet, we compiled GenBank accessions for ICTV VMR *Caudoviricetes* exemplars and additional isolates (5,822, of which 5,821 were available in GenBank), then compared this list against all accessions in the NCBI Viral RefSeq (v.230) database, removing duplicates. This identified 311 *Caudoviricetes* phages that represent known species in ICTV’s database and were not yet captured in Viral RefSeq. We concatenated these 311 sequences with the genomes of TayeBlu and the three NCBI nr relatives to build a non-redundant set for comparison against Viral RefSeq (v.230). We used vConTACT3 with default parameters to perform protein clustering and network-based classification leveraging the ICTV framework. Final taxonomic assignments were determined based on the optimal distance thresholds identified for each taxonomic rank.

### Comparative genome and core gene analysis

TayeBlu, the three best BLAST hits from NCBI nr, and the five other Viral RefSeq phages of novel_family_3 were analyzed for genome comparison and core gene identification. In order to perform these analyses, we downloaded the FASTA files of all eight phages from NCBI and performed genome annotation using pharokka with the -g prodigal-gv option to ensure consistent annotation strings (75, 89). The resulting GenBank files, together with our manually curated TayeBlu .gbk file, were analyzed and visualized for gene cluster synteny using clinker genome analysis with the default minimum alignment sequence identity (0.3) (90). Core gene analysis was performed by clustering predicted protein sequences using MMseqs2 with a minimum sequence identity threshold of 30% and a coverage threshold of 80% (–min-seq-id 0.30 -c 0.8). The protein cluster results and GenBank annotations were further analyzed in R (v.4.4.3) with dplyr, tidyr, and stringr (all tidyverse v.2.0.0) for data wrangling, then visualized with ggplot2 (v.3.5.2), scales, and patchwork (42, 43, 48, 91). Clusters with representatives from all nine analyzed phages were considered core genes; those with representatives from eight of the nine were considered near-core.

### Comparison with PIGEON database environmental phages

We downloaded 515,763 metagenomic viral operational taxonomic units (vOTUs) from natural soil and rhizosphere environments from the PIGEONv2.0 database (https://datadryad.org/stash/dataset/doi:10.25338/B8C934, accessed May 2025 [92]). This data set includes 53,391 vOTUs specifically derived from the tomato rhizosphere, an environment similar to the sample from which TayeBlu was isolated, among 192,008 vOTUs from a range of soil environments. Species-level clustering of TayeBlu with the complete PIGEONv2.0 database was performed using CheckV (v.0.8.1) and its associated scripts, anicalc.py and aniclust.py (64), with a threshold of 95% ANI over 80% genome coverage. To comprehensively assess TayeBlu’s relationship to soil viral diversity, we performed protein clustering analysis using vConTACT3 (v.3.1.3) (85) on the concatenated genome sequences of the nine phages of TayeBlu’s novel family and all 192,008 soil vOTUs in PIGEONv2.0. We examined the complete set of vConTACT3 taxonomic assignments to identify the vOTUs that cluster with TayeBlu at each taxonomic level.

## RESULTS AND DISCUSSION

### Isolation and morphological characterization of novel phage TayeBlu

**Fig 1:**
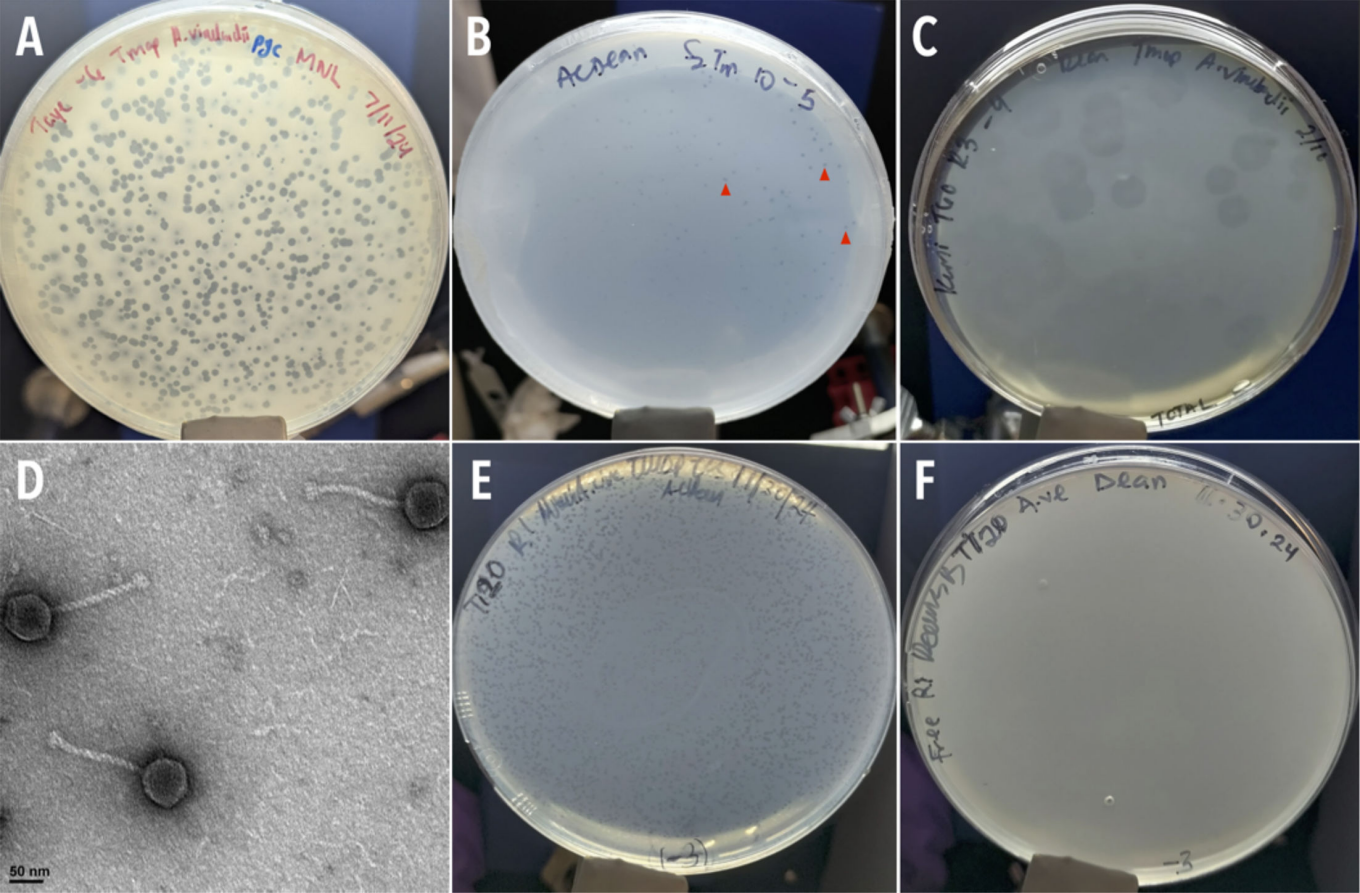
TayeBlu plaque and capsid morphology. (**A**) Plaques on *A. vinelandii* strain OP on rich medium (PYCa). (**B and E**) Plaques on N-replete minimal medium (AC_Dean). Red arrowheads in panel B mark representative plaques. (**C and F**) Variable plaques on minimal medium (Dean), with some infections yielding large temperate-like plaques (**C**) and others yielding no discernible plaques (**F**). Plates in panels **E** and **F** were inoculated in parallel from the same phage stock, with the plate in panel **E** serving as a positive control for phage stock infectivity. (**D**) Negative-stained transmission electron micrograph of phage particles.

TayeBlu was successfully isolated from soil samples collected from the greenhouse farm enclosure at the CWRU University Farm (Hunting Valley, OH). As an initial investigation of the effects of nitrogen availability on infection, we asked whether plaque morphology differed across media. We consistently observed the clearest plaques on rich medium (PYCa, diameter ∼1.6 mm; Fig. 1A) and tiny plaques on minimal medium amended with ammonium chloride as a nitrogen source (AC_Dean, ∼0.6 mm; Fig. 1B and E). Plaques on minimal medium (Dean) varied, with some plates showing temperate-like plaques (∼7.2 mm, Fig. 1C) and others showing no clear plaques, though parallel inoculation of AC_Dean plates confirmed the presence of active phage in the inocula (Fig. 1F; compare to panel E). Morphological characterization by TEM revealed an icosahedral head with a mean diameter of 67 ± 3 nm (*n* = 15) and a long, non-contractile tail (mean length 148 ± 6 nm, *n* = 11; Fig. 1D), features historically associated with siphoviruses (93).

### Infection dynamics

**Fig 2:**
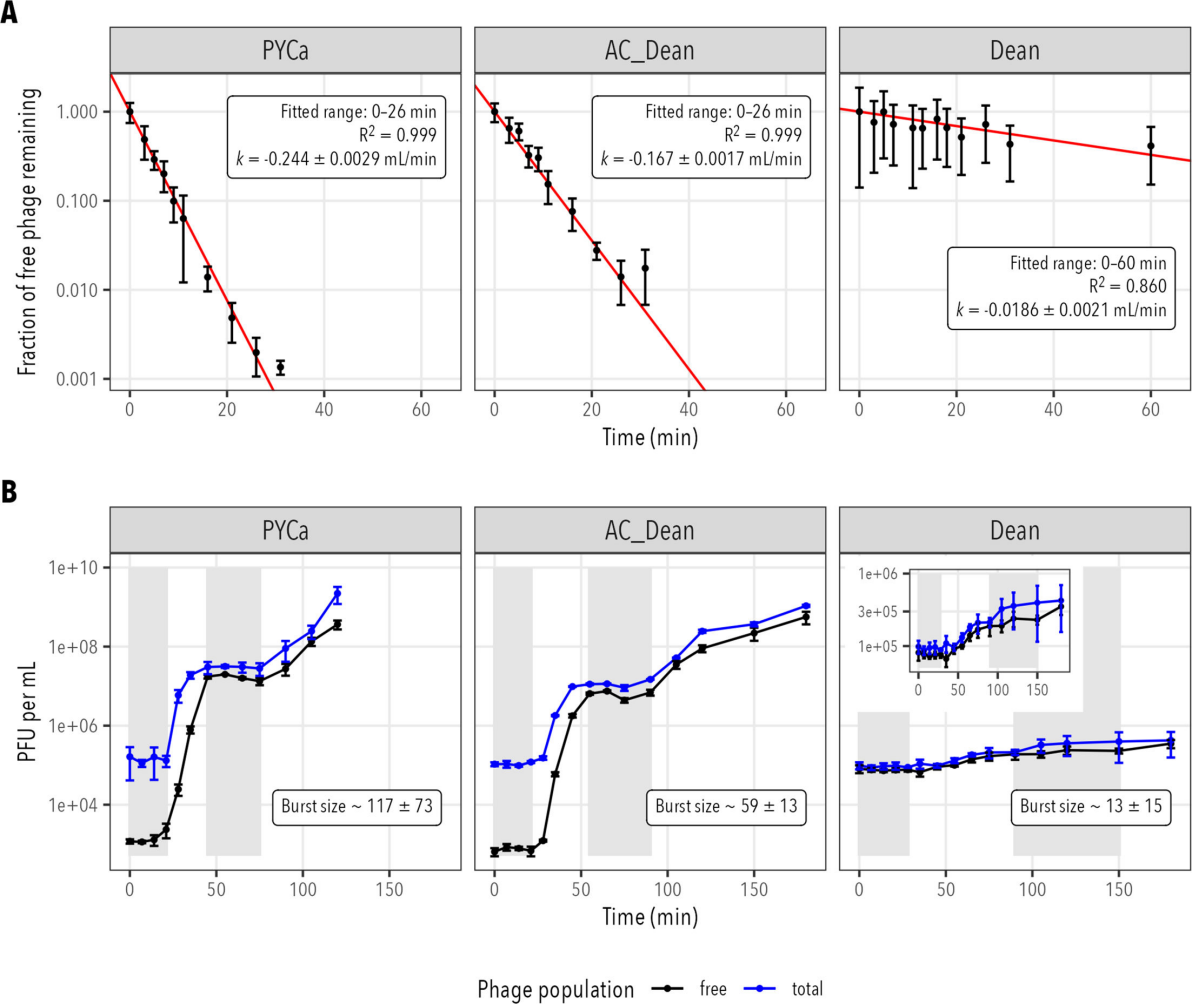
TayeBlu infection physiology on *A. vinelandii* strain OP, in rich (PYCa), N-replete minimal (AC_Dean), and minimal (Dean) media. Each datapoint represents the mean of three to six independent measurements, weighted by Poisson error estimates; error bars show the standard deviation of the weighted mean. (**A**) Adsorption kinetics. Red lines show linear fits to natural log-transformed data in the stated range. Values for k are fitted slopes ± standard error. (**B**) One-step infection curves (MOI ~0.1). Burst size estimates (weighted mean ± standard deviation) are based on the regions highlighted in gray for each medium (see Materials and Methods).

Phage TayeBlu infection dynamics on *A. vinelandii* strain OP in liquid medium, like its plaque morphologies on solid medium, were significantly influenced by media composition, with effects spanning the entire infection cycle. TayeBlu’s adsorption rate constant and total extent of adsorption were both lower by an order of magnitude in minimal medium than in rich medium (Fig. 2A). Ammonium amendment of the minimal medium substantially rescued adsorption, although it remained significantly slower than in rich medium (*P* = 7.7 × 10^−12^, Welch modified two-sample *t*-test). Similarly, bursts were both later (first clear evidence of burst, 55 min vs 28 min) and far smaller (burst size 13 ± 15 vs 117 ± 13, *P* = 1.119 × 10^−5^, Welch modified two-sample *t*-test) in minimal medium than in rich medium (Fig. 2B); ammonium amendment of minimal medium produced burst dynamics much closer in timing (first clear evidence of burst, 35 min vs 28 min) and scale (burst size 59 ± 13 vs 117 ± 73) to those in rich medium, though the difference in burst size remained significant (*P* = 0.003717).

The extent of rescue by ammonium amendment (AC_Dean) of minimal medium strongly suggests that TayeBlu infection dynamics in minimal medium are shaped primarily by the requirement for diazotrophy in these conditions. Previous studies have shown that nutrient availability can modulate the expression of outer membrane proteins and lipopolysaccharides that commonly serve as phage receptors (94, 95). In addition to any such changes, *Azotobacter* in well-aerated minimal medium is expected to grow diazotrophically and to deploy nitrogenase-protective mechanisms that are likely to alter phage infection. First, under N-fixing conditions, many *Azotobacter* strains substantially increase production of the extracellular product alginate, thought to limit diffusion of O_2_ from the medium to the cell (96). Although *A. vinelandii* strain OP is generally considered a “non-gummy,” alginate non-producing strain due to a spontaneous loss of function in its *algU* gene (97), we have frequently observed that a gummy phenotype spontaneously re-emerges during propagation under N limitation (Fig. S2); if present, an alginate capsule or other extracellular polysaccharide layer could easily alter receptor availability for phage, hampering adsorption (98). Second, *Azotobacter* greatly increases its respiration rate to draw down intracellular O_2_, creating high C-substrate demand under N limitation (31, 99, 100). In minimal medium, this high respiration rate could compromise resource availability for phage reproduction, extending the latent period and limiting burst size. The large change to infection dynamics observed here between rich and minimal media demonstrates the potential variability of TayeBlu impacts across soils with varying nutrient availability, highlighting the importance of characterizing soil phage infection dynamics across a range of growth conditions to support quantitative modeling of ecosystem impacts (101).

### Genome analysis

Genome sequencing (median coverage: 2,191) revealed that TayeBlu possesses a double-stranded DNA genome of 59,885 bp with a G + C content of 50.34% (Table 1). The assembly was circular, as expected for completely sequenced dsDNA phage with linear genomes and terminal repeats or circularly permuted genomes. Coverage analysis identified a 5′ terminus on the forward strand, just upstream of the genes encoding the small and large terminase proteins, and no clear 3′ terminus, consistent with the pattern expected for headful packaging (65, 102). Phylogenetic analysis of the large terminase protein with those of phages of known packaging mechanism (103, 104) confirmed that TayeBlu’s terminase clusters with those of known headful packaging phages (Sf6 group, Fig. S3).

**TABLE 1:** Genomic features of bacteriophage TayeBlu

Genomic feature	Value
Genome size (bp)	59,885
G + C%	50.34
Coding %	94.76
CDS	100
tRNAs	0
Transcription terminators	15–18
Start codons	
ATG	86
GTG	12
TTG	2

**Fig 3:**
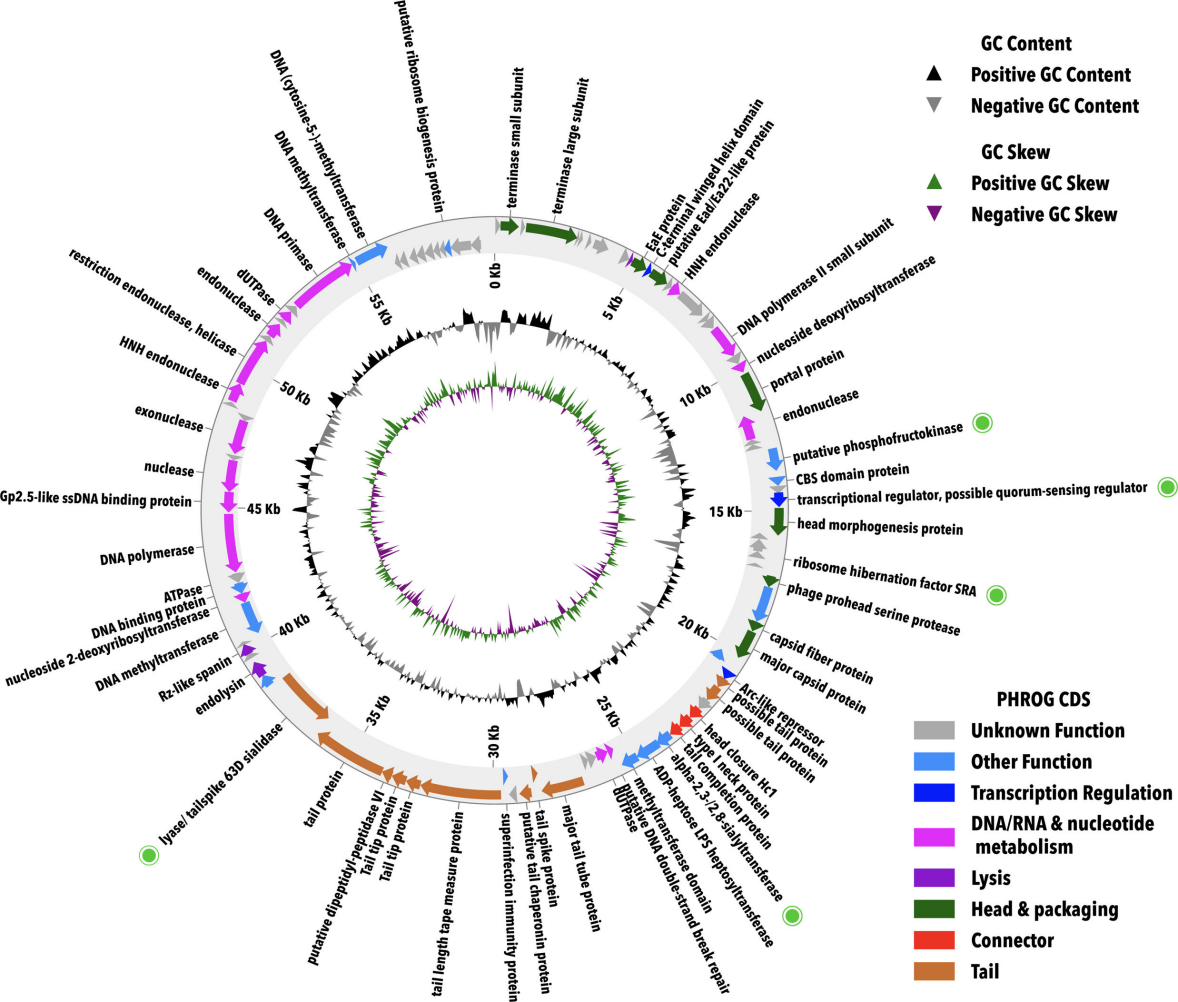
Complete circular genome of novel phage TayeBlu. Manually curated gene annotations (arrows, outermost rings) are colored by PHROG functional category. Nucleotide-level genome composition patterns are shown in the middle (GC content) and inner (GC skew) rings. Genes with potential functional significance discussed in the text are marked with light green bullets.

Structural and functional annotation using automated tools followed by manual curation in Apollo predicted 100 CDSs with no identifiable tRNA genes. We identified 15–18 putative transcription terminators from the 30 predicted using ARNold (81) (Table 1; Table S1). Start codon usage showed a predominance of ATG, with lesser usage of GTG and TTG. Functional annotation of the genome classified the CDSs into several categories (Fig. 3): structural and assembly genes (23 genes, comprising head and packaging proteins, connector proteins, and tail proteins), DNA, RNA, and nucleotide metabolism (16 genes), lysis (3 genes), transcription regulation (3 genes), and other functions (14 genes), while the rest of the genes encode hypothetical proteins (41) with no known function (Fig. 3).

### Genes with potential functional significance

Intriguingly, TayeBlu encodes a putative phosphofructokinase (locus AZP_TayeBlu_0026), previously reported as an AMG in marine and gut viruses (13, 105). This enzyme catalyzes the conversion of fructose-6-phosphate to fructose-1,6-bisphosphate, an ATP-consuming early step in the Embden-Meyerhof-Parnas (EMP) glycolytic pathway that is the point at which a substrate glucose molecule is energetically committed to the pathway. Thus, any phage-mediated expression of phosphofructokinase during infection should partition glycolytic flux toward this pathway and away from the competing Entner-Doudoroff (ED) pathway. Fluxomic analysis of *A. vinelandii* has shown that only a small minority of glycolytic flux in uninfected cells uses EMP, while the bulk is directed through ED despite its lower ATP yield (99). Hypothesizing that the trade-off lowers the N demand for glycolytic enzyme synthesis, Wu et al*.* argue that *A. vinelandii* tunes central C metabolism so that the redox state of the cell supports respiratory protection of nitrogenase (99). Phage-mediated rebalancing of glycolytic flux might thus put nitrogenase at risk. We hypothesize that TayeBlu will express phosphofructokinase during infection under conditions where phage replication is limited more by ATP than by N availability. Under this hypothesis, TayeBlu could not only directly influence carbon use efficiency but also indirectly influence host nitrogen fixation despite the absence of recognized nitrogen-cycling AMGs.

Beyond central metabolism, several genes annotated in TayeBlu may influence phage-host interactions. Putative carbohydrate-active enzymes include a lyase/tailspike with 63D sialidase homology (locus AZP_TayeBlu_0064) and an alpha-2,3-/2,8-sialyltransferase (AZP_TayeBlu_0047), which might interact with *A. vinelandii*’s exopolysaccharides, including alginate. Viral sialidases often facilitate cell entry or exit by modifying surface glycans (106); earlier work has demonstrated alginate lyase activity in an unsequenced *A. vinelandii* phage (36). Additionally, we identified a putative paratox (Prx) domain protein (AZP_TayeBlu_0029) with sequence similarity to inhibitors of the quorum sensing receptor ComR (107). Quorum sensing regulation plays a critical role in biofilm formation, e.g., by altering production of extracellular polymeric substances (EPS) including alginates (reviewed in reference 108); a paratox protein might allow TayeBlu to manipulate host cell signaling to alter biofilm formation or EPS production. Finally, the genome encodes a protein with homology to recently described ribosome hibernation factors (AZP_TayeBlu_0033) (109), which may influence host translation machinery under nutrient-limited conditions. The roles of these genes in the TayeBlu infection cycle across growth conditions remain to be experimentally validated.

### Taxonomic placement

Following current ICTV guidelines (93), we sought to classify TayeBlu on the basis of its genome (110). BLAST searches of all viruses in the NCBI Nucleotide collection (nr/nt) database against the complete TayeBlu genome revealed three moderately similar phages with non-trivial query coverage: Enterobacter phage Mulvp2 (OR508996.1; 67% query coverage, 93.93% identity), Caudoviricetes sp. ctumj2 (BK048414.1; 64% query coverage, 93.78% identity), and Siphoviridae sp. ctdc_1 (MH622927.1; 26% query coverage, 72.38% identity). Next, we used the proteome clustering tool vConTACT3 (85) with Viral RefSeq (v.230) to place TayeBlu in realm *Duplodnaviria*, phylum *Uroviricota*, and class *Caudoviricetes*, consistent with its identification as a tailed bacteriophage (110). This analysis identified four additional phages with family-level similarity to TayeBlu: Klebsiella phage vB_KpnS-Carvaje (56,858 bp; 97 proteins), Proteus phage VB_PmiS-Isfahan (54,836 bp; 96 proteins), Salmonella phage 9NA (52,869 bp; 80 proteins), and Salmonella phage vB_SenS_Sasha (53,263 bp; 80 proteins).

**Fig 4:**
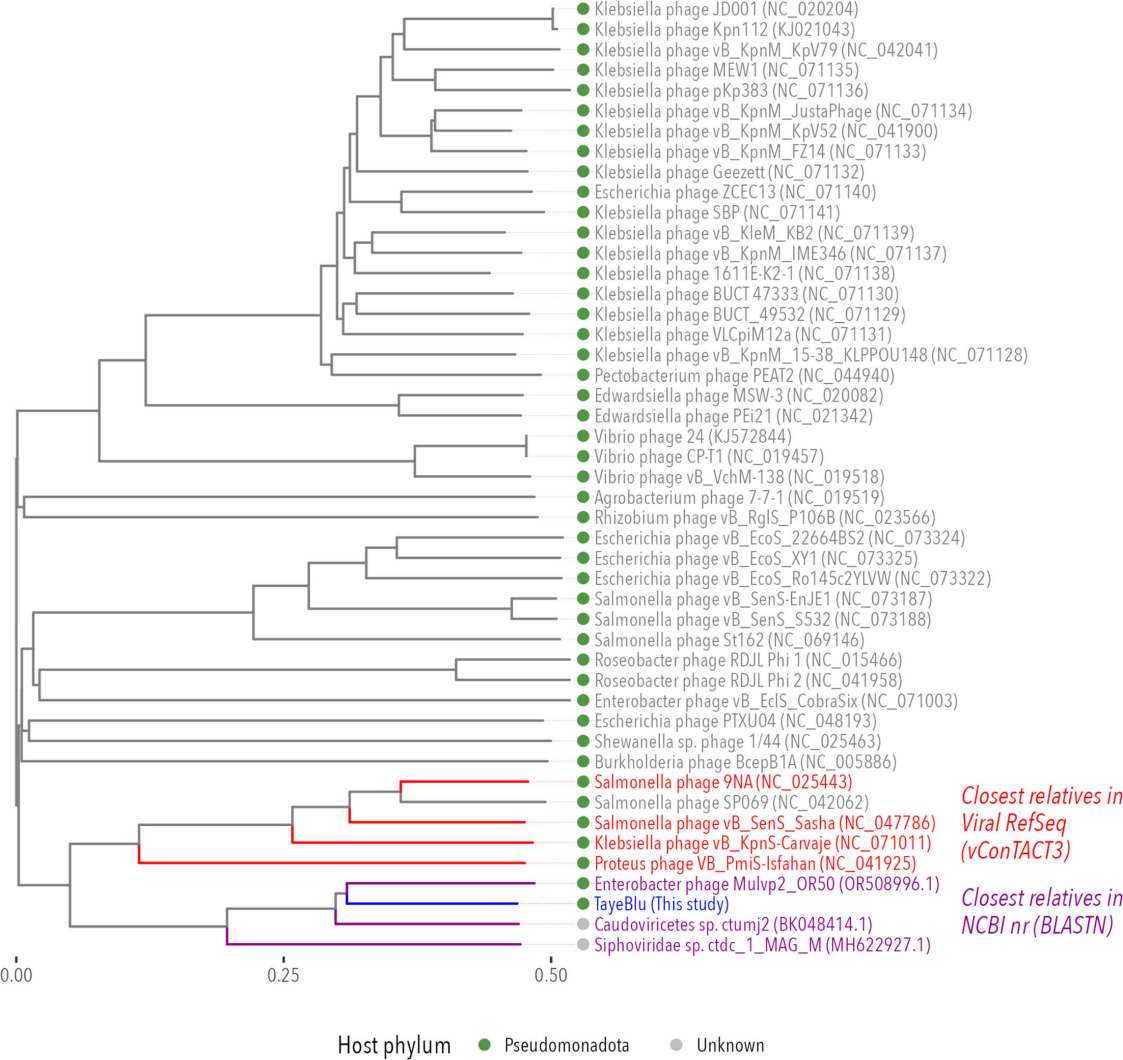
Proteome-based phylogenetic relationships between TayeBlu (blue), 3 close relatives identified in NCBI nr by genomic similarity (purple), and 43 additional relatives with ViPtree similarity scores of at least 0.020 (gray), including the 4 members of novel_family_3 identified by vConTACT3 (red). Nearly all viruses in the tree are known to infect hosts from phylum Pseudomonadota (green dots); the remaining two phage genomes were recovered from metagenomic sequencing, and their hosts are unknown (gray dots).

Next, we scored protein similarity to phages in the Virus-Host DB (111), identifying 43 phages (including the four family-level relatives identified by vConTACT3, but excluding one short satellite phage) with moderate similarity (ViPTree genomic similarity score ≥0.020) to TayeBlu, for a total of 47 at least moderately similar relatives (Table S2). In this expanded group, all phages whose hosts are known infect hosts within the phylum *Pseudomonadota*. A ViPTree proteome-based phylogenetic tree for TayeBlu, its three NCBI relatives, and all phages in Virus-Host DB revealed that TayeBlu forms a single, cohesive monophyletic lineage with the seven close relatives identified above and an additional *Salmonella* phage, SP069 (TayeBlu and moderately similar relatives, Fig. 4; full tree, Fig. S5). This proteome tree demonstrates that a significant number of orthologous genes are shared among TayeBlu and its eight close relatives, consistent with ICTV family-level classification standards.

To refine TayeBlu’s taxonomic placement, we sought to compare it against all established *Caudoviricetes* diversity, as captured by ICTV’s Virus Metadata Resource. We repeated the vConTACT3 analysis, clustering the members of the TayeBlu ViPTree family with the ICTV *Caudoviricetes* species exemplars and additional isolates, as well as the rest of Viral RefSeq (v.230). TayeBlu clustered with 5,378 *Caudoviricetes* viruses, with 534 of these in novel_order_21. No additional ICTV phages were assigned to TayeBlu’s family, novel_family_3 (Fig. S4; Table S3), indicating that this is a previously uncharacterized lineage within *Caudoviricetes*. Two of the four phages in TayeBlu’s novel_subfamily_0 are known only from metagenomic sequence. By contrast, several phages of the 9NA group in sister clade novel_subfamily_1 have been characterized experimentally, and the members of this clade have been shown to be only distantly related to other known Siphoviridae (104). We propose that these two subfamilies constitute a novel phage family.

**Fig 5:**
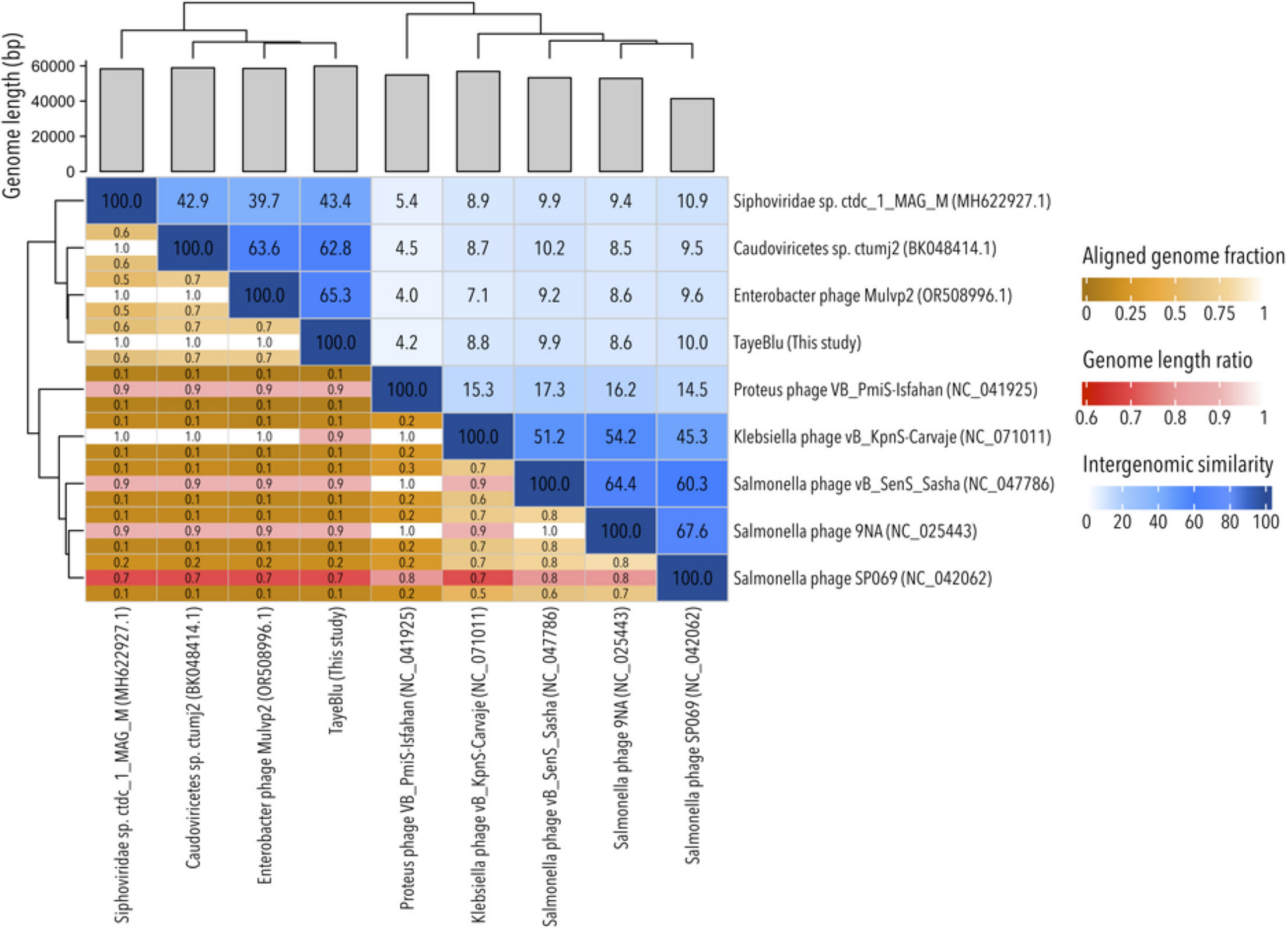
VIRIDIC intergenomic similarity matrix for TayeBlu and the other identified members of the novel family highlighted in this study.

Analysis of novel_family_3 at the nucleotide level suggests substantial diversification both between and within the two subclades evident in the proteome tree. As expected, VIRIDIC intergenomic similarity analysis showed that the closest relative to TayeBlu was its top BLAST hit, sharing only 65.3% ANI (Fig. 5). Notably, TayeBlu exhibited minimal nucleotide similarity (≤10%) with the ViPTree- and vConTACT3-identified family members in the sister subclade. This observation is consistent with the growing recognition that phage taxonomy should rely primarily on protein conservation patterns, as the extent of diversification among related phage can obscure relationships at the nucleotide level (112, 113) and, further, that family-level groupings represent broader genetic diversity among bacteriophage than among eukaryotic viruses (114). With current ICTV intergenomic similarity thresholds of ≥95% for the species level and ≥70% for the genus level, the highest intergenomic similarity observed here (67.6%) falls short of the genus-level threshold. However, proteome-based clustering places the uncharacterized Enterobacter phage Mulvp2_OR50 and Caudoviricetes sp. ctumj2 in the same genus (novel_genus_0) as TayeBlu, a discrepancy that reflects well-documented challenges in viral taxonomy (115). Whether or not continued improvements in phage taxonomy conclusively show these three phages to be congeneric, TayeBlu is the first phage of its genus and species to be characterized physiologically.

### Genome comparison and core gene identification

**Fig 6:**
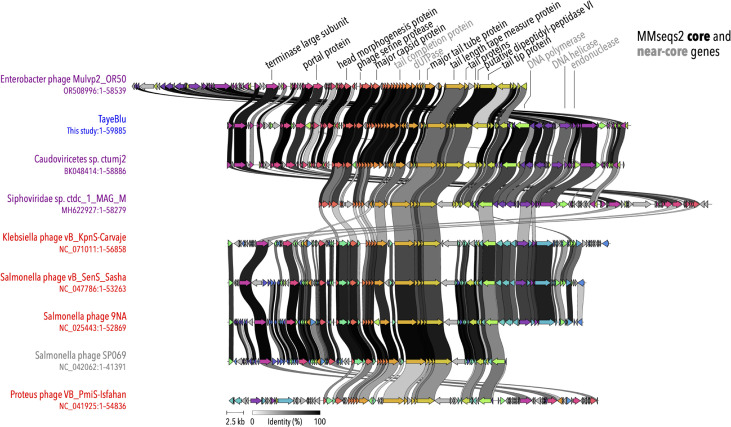
Core genes and synteny relationships conserved across the two subfamilies of novel_family_3. Sequence names are colored as in Fig. 4: blue, TayeBlu; purple, phage identified by BLASTN in NCBI nr/nt; red, phage identified by vConTACT3 in Viral RefSeq; gray, phage identified by ViPTree in the Virus-Host DB. Ribbons connect shared genes identified by Clinker alignments; ribbon shading is scaled to pairwise percent identity at the protein level. Core genes (gene functions labeled in black) were identified by MMseqs2 protein clustering as present in all phage and near-core genes (gray) as present in eight of nine phages.

TayeBlu and its eight identified relatives have genomes of 41–59 kb with 69–100 predicted open reading frames (ORFs) (Fig. 6), a total of 799 ORFs across the family. We applied protein clustering (sequence identity ≥30%, coverage ≥80%) to identify this novel family’s core genes, finding 397 unique clusters (Table S4; Fig. S5) of which 11 were present in all 9 examined genomes. These 11 core genes, ∼12% of the total ORFs in this phage family, constitute the family’s conserved genetic framework. An additional five genes were shared between TayeBlu and all but one phage in the family, extending the near-core genome to 16 genes. Functional annotation of these conserved genes revealed that they primarily encode structural components (capsid and tail proteins), DNA packaging machinery (terminase and portal proteins), and enzymes involved in DNA replication (DNA helicase, polymerase, and dUTPase), alongside an endonuclease. Genome regions encoding structural tail proteins and DNA packaging machinery also show substantial synteny (Fig. 6).

The predominance of structural and assembly genes in the core genome is consistent with previous observations that these functional categories tend to be the most highly conserved genes across diverse phage lineages (116, 117). The critical importance of these functions in the phage life cycle likely places these genes under higher selective pressure than the rest of the genome, as seen previously in tailed bacteriophage large terminase genes (reviewed in reference 103). The limited but syntenic core genome suggests that the essential framework for virion assembly and DNA replication has remained intact during adaptive radiation to the family’s ecological niches. Notably absent from the core genome are genes involved in transcriptional regulation and host takeover functions (Fig. S6), suggesting diverse host interaction strategies. The conservation pattern observed in this novel phage family, with structural proteins, DNA packaging components, and replication machinery forming the stable core within a family marked by low overall genome similarity, is consistent with the observation that phages can maintain a stable core genome over extended periods while acquiring variable complements of genes through recombination from the phage pan-genome (118), highlighting the balance between functional conservation and adaptive diversification.

### Clustering with phages isolated from natural soil and rhizosphere

Finally, we investigated TayeBlu’s genomic relatedness to vOTUs from environmental sampling, as collected in the PIGEONv2.0 database (92), using clustering based on average nucleotide identity and gene-sharing networks. In the complete database of 515,763 environmental vOTUs, no sequences clustered with TayeBlu at species-level thresholds (ANI ≥95% with ≥80% of sequences aligned). Protein-based clustering of the nine phages in TayeBlu’s family with the 192,008 vOTUs from soil environments found 56,968 vOTUs in class *Caudoviricetes*, but of these only a single environmental vOTU, sampled in a Northern California saltwater wetland (119), clustered with TayeBlu at the family level (see Table S5 at https://zenodo.org/records/16696721). Together, these analyses indicate that TayeBlu represents a novel viral lineage. Soil viromes are known to harbor extensive genetic novelty (120, 121), and this vast diversity is still largely unexplored (122). Even across different rhizosphere habitats, phages display exceptionally diverse communities depending on plant species, soil type, and geographic location (122–124), with most identified viral sequences showing little similarity to known phages from any environment (123). This suggests highly specialized adaptation to ecological niches that are subject to plant-soil-microbe interactions. Strikingly, the environmental vOTUs examined here include 53,391 from the tomato rhizosphere, like TayeBlu itself. The lack of phage genomes closely related to TayeBlu even within this set underscores the limited representation of soil viral diversity in current databases and the pressing need for expanded efforts to catalog and study phages from diverse terrestrial ecosystems.

### Conclusion

The novel phage TayeBlu, isolated on *Azotobacter vinelandii* strain OP from agricultural rhizosphere soil, is a member of a novel family and represents a distinct genus and species within this viral lineage. Its infection physiology varies dramatically depending on nutrient availability and particularly on the exogenous supply of fixed nitrogen to its facultatively diazotrophic host. We hypothesize that this variation will alter the phage’s ecological impacts across different soils and agricultural practices, potentially influencing bacterial community structure and evolution (10, 125), N flow within the rhizosphere, and ecosystem outputs from the globally significant process of biological N fixation. Genomic analysis reveals extensive diversification even among the few members of this phage family, highlighting the need for future isolation efforts to illuminate the ecological roles and impacts of phage in soil systems.

## Data Availability

The complete genome sequence of phage TayeBlu has been submitted to GenBank with accession number PV565308. Code for bioinformatic analysis is publicly available at https://github.com/Taiwomercy/TayeBlu_pub. Table S5 is available at Zenodo (doi: 10.5281/zenodo.16696721).

## SUPPLEMENTAL MATERIAL

Supplemental figures spectrum.01286-25-s0001.pdf Fig. S1 to S6.

Table S1 spectrum.01286-25-s0002.xlsx Transcriptional terminators identified in phage TayeBlu.

Table S2 spectrum.01286-25-s0003.xlsx ViPTree proteome similarity scores.

Table S3 spectrum.01286-25-s0005.csv vConTACT3 protein-based phylogenetic clusters.

Table S4 spectrum.01286-25-s0004.csv MMseqs2 protein-level clusters.

ASM does not own the copyrights to Supplemental Material that may be linked to, or accessed through, an article. The authors have granted ASM a non-exclusive, world-wide license to publish the Supplemental Material files. Please contact the corresponding author directly for reuse.
