# Supplementary material for: Isolation and characterization of TayeBlu, a novel bacteriophage of Azotobacter vinelandii
Source: Microbiol Spectr. 2025 Dec 9;14(1):e01286-25. doi: 10.1128/spectrum.01286-25 (PMC12772287; doi:10.1128/spectrum.01286-25)
Supplement: Supplemental figures — Fig. S1 to S6. [file spectrum.01286-25-s0001.pdf]

Supplementary Figures

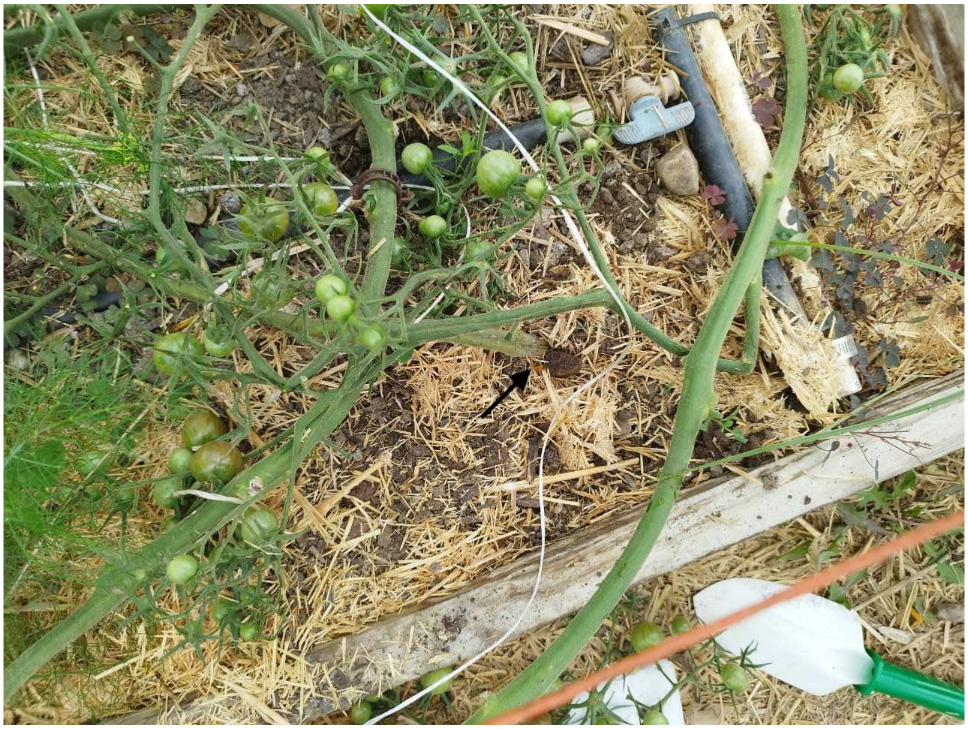

FIG S1 Tomato plant at the Case Western Reserve University Farm greenhouse prior to rhizospheric soil collection. Soil was sampled from the root-adjacent zone at the plant base (black arrow) using a sterilized hand shovel, targeting areas with visible fine roots.

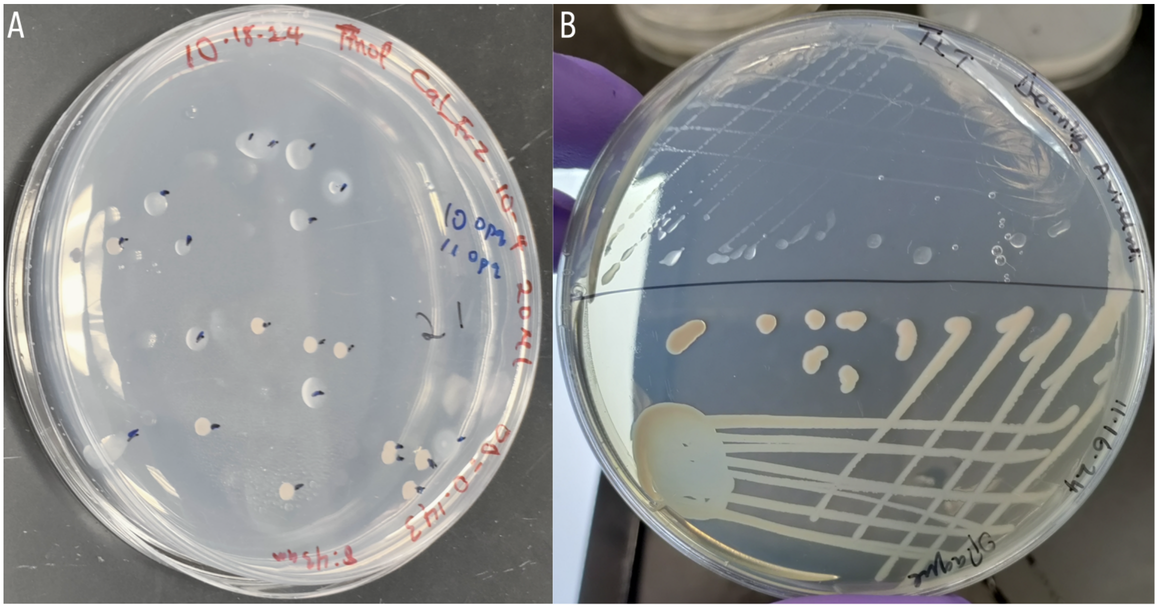

FIG S2 Phenotypic variation in *Azotobacter vinelandii* strain OP culture after growth in Dean liquid medium. (A) Spread plate of *A. vinelandii* strain OP culture on Dean solid medium at 10^{-4} dilution showing two distinct colony morphologies: gummy (larger, translucent, pearlescent) and non-gummy (compact, opaque, greenish-white) variants. (B) Streak plate on Dean solid medium demonstrating the isolated gummy (top) and non-gummy (bottom) morphotypes.

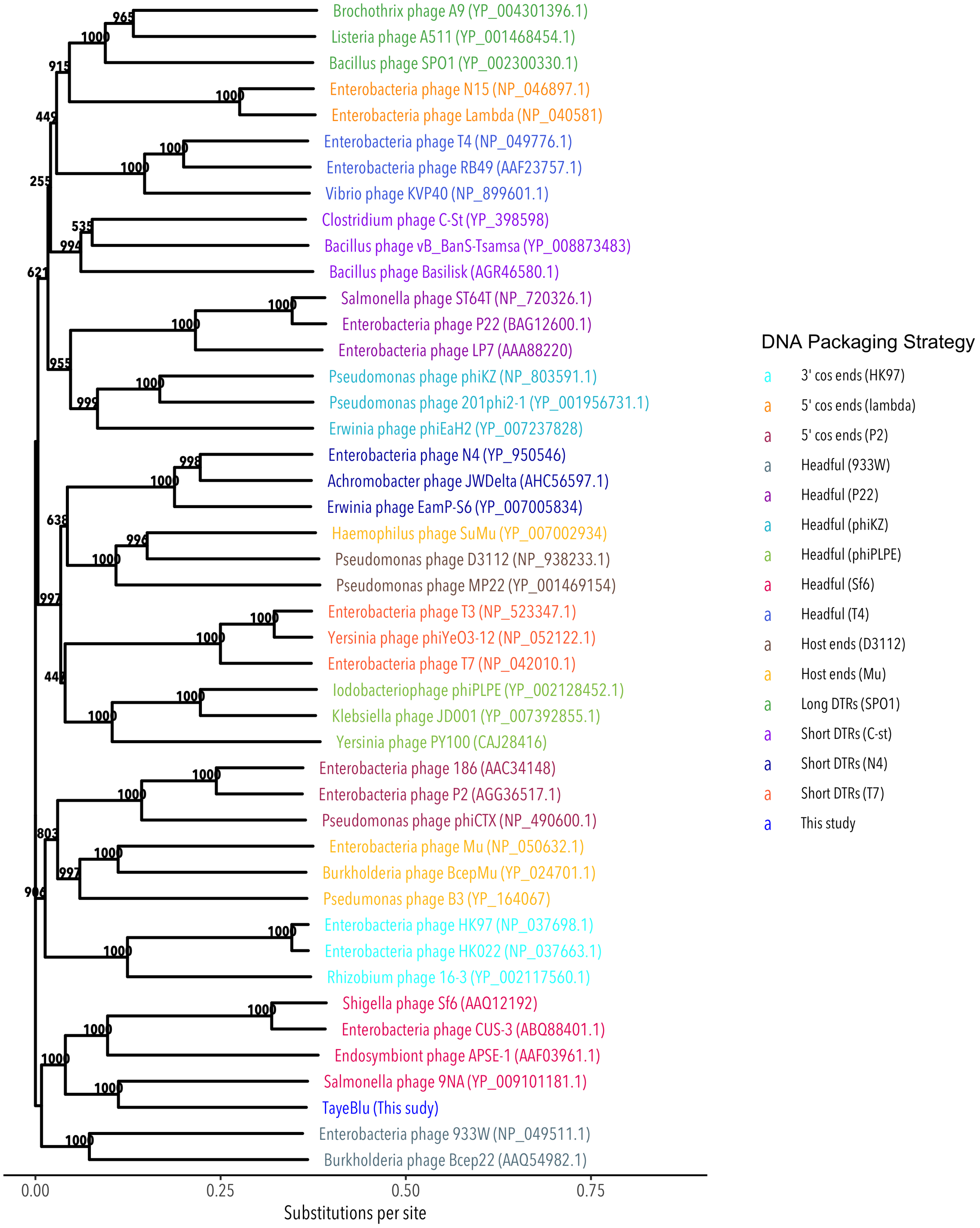

FIG S3 Neighbor-joining tree of large terminase protein sequences from TayeBlu and phages with experimentally characterized DNA packaging strategies. Reference sequences are as used in [103], with the addition of the large terminase from Salmonella phage 9NA, which has been shown to use headful packaging [104] and which vConTACT3 identified as highly similar to

TayeBlu. TayeBlu large terminase (blue) clusters with the large terminases of 9NA and phages known to employ the Sf6 headful packaging strategy (fuchsia).

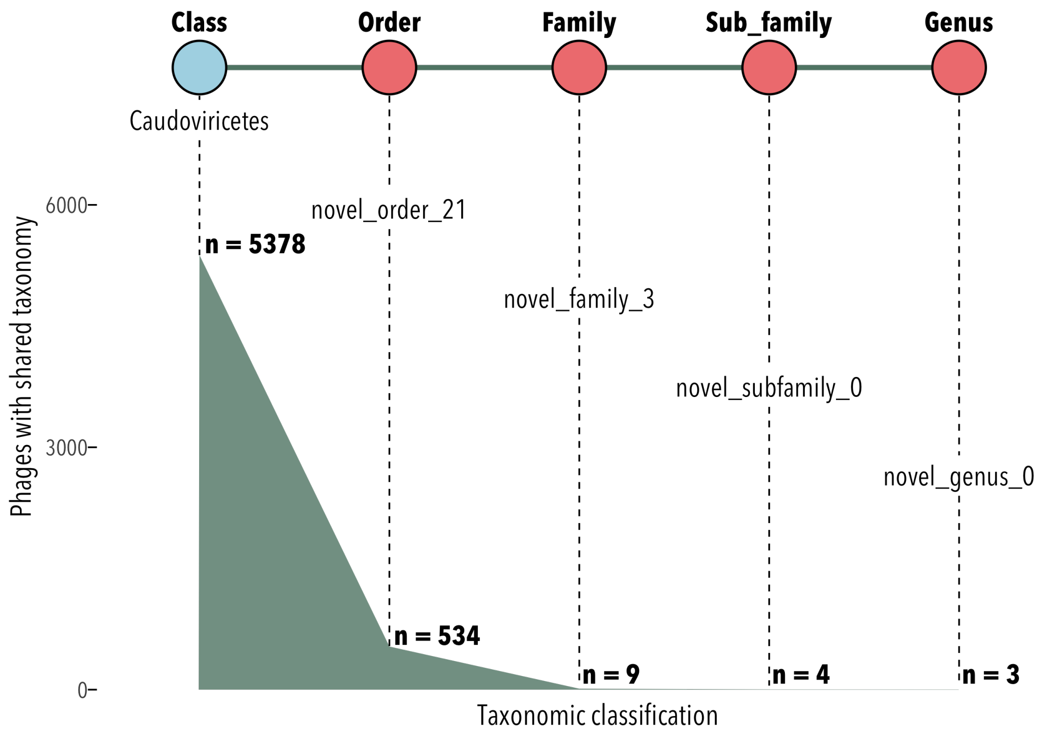

FIG S4 Taxonomic classification of TayeBlu by vConTACT3, against a comparison set of Viral RefSeq v230, all other ICTV Caudoviricetes exemplars, and the three phages identified by BLASTN in NCBI nr. The y-axis shows counts of phage in this dataset that share TayeBlu's taxonomy at each level. Counts include TayeBlu.

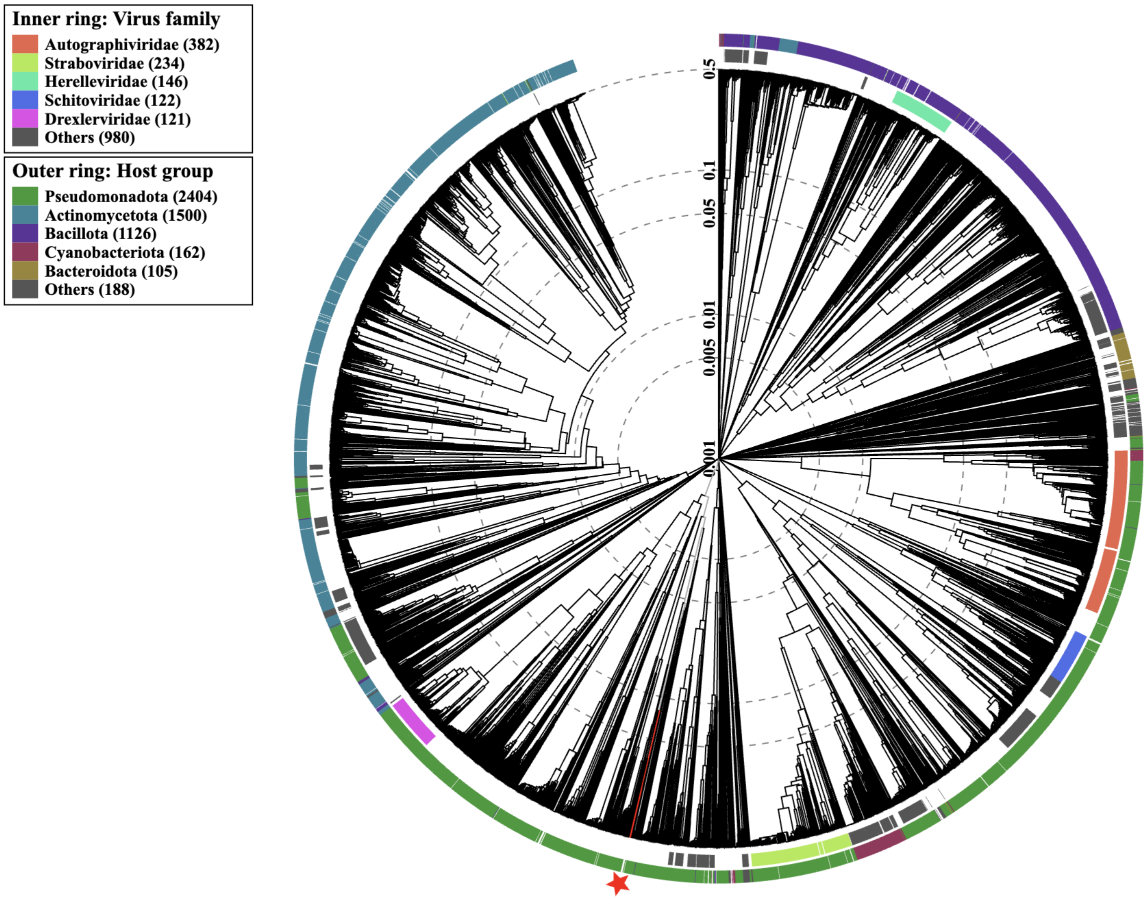

FIG S5 Protein-based phylogeny of all TayeBlu relatives identified by ViPTree in Virus-Host DB, showing viral families (inner ring) and host group (outer ring). TayeBlu is marked by a red star. TayeBlu and the four phage identified by vContACT3 are indicated by red branches.

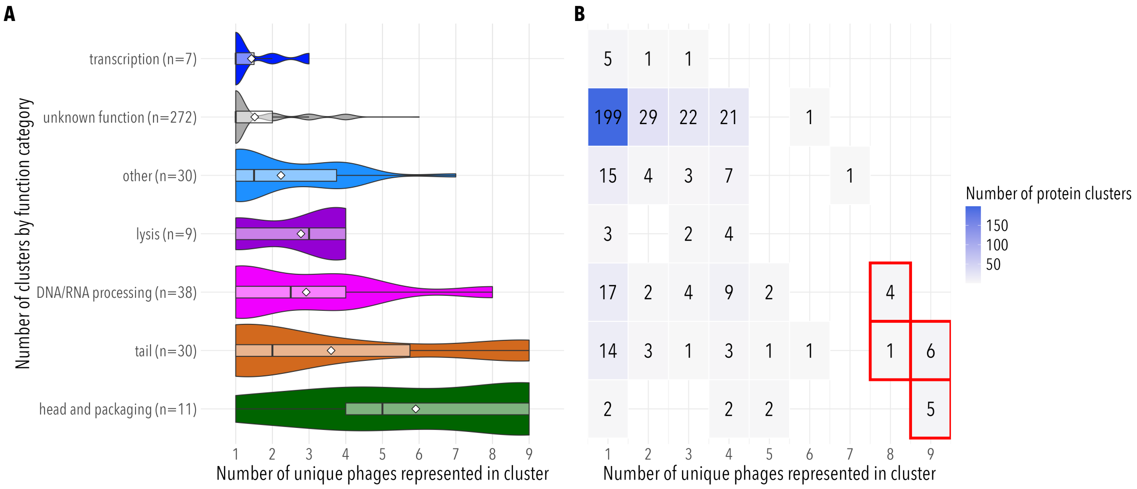

FIG S6 Distribution of protein cluster membership across the phage family by functional category. (A) Boxplot and violin plot. Mean, white diamonds; median, crossbar; lower bound, 25th percentile; upper bound, 75th percentile. Functional categories are ordered by mean number of phages represented in per cluster. (B) Heatmap of cluster counts. Data is the same as in (A). Clusters boxed in red contain core and near-core genes.
